# Inclusive dementia research: A guidebook to support researchers engaging with people living with dementia from ethno‐racial communities

**DOI:** 10.1002/alz70858_101677

**Published:** 2025-12-25

**Authors:** Carrie A McAiney, Navjot Gill‐Chawla, Emma Bender, Dana Zummach, Laura Knowlton, Christine Pellegrino, the Dementia & Diversity Steering Committee

**Affiliations:** ^1^ University of Waterloo, Waterloo, ON, Canada; ^2^ Schlegel‐UW Research Institute for Aging, Waterloo, ON, Canada

## Abstract

**Background:**

Despite the growing ethno‐racial diversity of people living with dementia globally and nationally, individuals who participate in dementia research often do not reflect this diversity. An environmental scan of resources that address the intersection of dementia, diversity and research failed to identify resources to support researchers interested in working in this area. A lack of guidance for researchers could result in tokenism, insensitive research practices, and further harm to ethno‐racial communities. The objective of this presentation is to describe the “Inclusive Research Guidebook: Partnering with People Living with Dementia from Ethno‐racial Communities”, a new resource that was co‐designed for researchers and research teams to support them in conducting research with ethno‐racial communities.

**Method:**

A diverse Steering Committee that included people living with dementia, family/friend care partners, providers working with people living with dementia and/or people who are part of ethno‐racial communities, and researchers was assembled. Over a 1.5‐year period, the group worked together to co‐design the Inclusive Research Guidebook. Development of the Guidebook included: a rapid review of the literature; monthly meetings with the Steering Committee to develop and review draft content for the Guidebook; an in‐person symposium involving a group of 35 people living with dementia, care partners, providers and researchers to review and provide feedback on the Guidebook using a World Café process; and incorporation of the feedback and further engagement with the literature.

**Result:**

The Guidebook provides information, reflective questions, and resources to support researchers and research teams in considering: (1) individual biases, assumptions, and privileges; (2) how to learn about the community the researchers want to work with, including the community's identity, history, and trauma; (3) building relationships and trust with communities; and (4) considerations for the research team and research processes. The presentation will highlight insights into the co‐design process and the key concepts addressed in the Guidebook.

**Conclusion:**

It is anticipated that the Guidebook will help to promote safe and inclusive research with ethno‐racial communities.

